# Mechanism of Elevated CO_2_ Delaying Senescence of Postharvest *Agaricus bisporus* by Regulating Energy Metabolism: Insights from Metabolomics

**DOI:** 10.3390/foods15122147

**Published:** 2026-06-14

**Authors:** Liyao Zhou, Wenying Tong, Jie Chen, Shun Yang, Donglu Fang, Ning Ma, Wenjian Yang, Qiuhui Hu, Fei Pei

**Affiliations:** 1 Jiangsu Province Engineering Research Center of Edible Fungus Preservation and Intensive Processing, Nanjing 210023, China; liyao0610z@163.com (L.Z.); tongwenying2023@163.com (W.T.); m18571879187@163.com (J.C.); 13293531266@163.com (S.Y.); maning@nufe.edu.cn (N.M.); lingwentt@163.com (W.Y.); qiuhuihu@nufe.edu.cn (Q.H.); 2 Collaborative Innovation Center for Modern Grain Circulation and Safety, College of Food Science and Engineering, Nanjing University of Finance and Economics, Nanjing 210023, China; 3 State Key Laboratory of Tree Genetics and Breeding, Co-Innovation Center for Sustainable Forestry in Southern China, College of Forestry and Grassland, Nanjing Forestry University, Nanjing 210037, China; fangdonglu@njfu.edu.cn

**Keywords:** metabolomics, glycolysis, TCA cycle, amino acid metabolism, purine metabolism

## Abstract

*Agaricus bisporus* (*A. bisporus*) is susceptible to rapid postharvest deterioration. Although elevated CO_2_ (6%) delays senescence, the metabolic mechanisms remain unclear. In this study, untargeted and targeted metabolomic analyses were employed to explore these pathways in *A. bisporus*. The results revealed that elevated CO_2_ treatment promoted glycolysis by upregulating Hexokinase (*HK*), Phosphofructokinase (*PFK*), and Pyruvate Kinase (*PK*), accumulating Glucose-6-phosphate (G-6-P) and Fructose-6-phosphate (F-6-P). Concurrently, elevated CO_2_ treatment upregulated the expression of genes associated with the tricarboxylic acid (TCA) cycle and increased the enzymatic activities of Malate Dehydrogenase (MDH) and Fumarate hydratase (FUM). These changes led to the rapid consumption of key intermediate metabolites (Fumarate (Fum), Malate (Mal), and α-Ketoglutarate (α-KG)), collectively enhancing the efficiency of the TCA cycle. Furthermore, elevated CO_2_ treatment significantly suppressed the activities of Glutamine Synthetase (GS) and Xanthine Oxidase (XOD), inhibiting the synthesis of Glutamine (Gln) and Pyroglutamate (pGlu) while promoting the accumulation of Hypoxanthine (Hx). This coordinated reprogramming of amino acid metabolism and purine metabolism contributed to improved energy efficiency and enhanced cellular integrity in postharvest *A. bisporus*. This study elucidates the specific mechanism by which elevated CO_2_ levels regulate the postharvest energy metabolism of *A. bisporus* from a metabolomics perspective, providing a theoretical basis for developing strategies to control its postharvest quality.

## 1. Introduction

*Agaricus bisporus* (*A. bisporus*), one of the most widely cultivated and consumed edible mushrooms globally, is highly valued for its rich nutritional profile, including proteins, polysaccharides, vitamins, and various bioactive compounds [1,2]. However, its high moisture content, vigorous respiration, and rapid metabolic activity postharvest lead to severe quality degradation. These deteriorations, including cap opening, browning, texture softening, and flavor loss, present major obstacles to its postharvest preservation and logistics [3]. To address these issues, a range of technologies has been developed and applied for *A. bisporus* preservation [4,5,6,7]. Among these, modified atmosphere packaging is widely recognized as a promising physical preservation technique, owing to its high efficiency, safety, and lack of chemical residues, and has been increasingly utilized to maintain the quality of this mushroom species [8].

As the cornerstone of life’s metabolism, abundant energy safeguards the intricate cellular dance of postharvest edible fungi, gracefully delaying their natural decline [9]. Maintaining energy homeostasis relies on a sophisticated metabolic system, primarily composed of coupled pathways such as Glycolysis and the tricarboxylic acid (TCA) cycle [10,11]. Once detached from the parent body, *A. bisporus* is prone to abnormal respiratory metabolism, leading to insufficient energy supply and metabolic pathway imbalances. Consequently, this triggers a series of quality degradation issues, including cellular structure disruption, enzymatic browning, and reduced antioxidant capacity [12,13]. Research has revealed that the root cause of these crises lies in dysfunctional energy metabolic pathways. Mitochondria, known as the cellular “powerhouses,” exhibit functional decline over storage time, directly resulting in reduced ATP synthesis [14]. This is followed by decreased efficiency in the TCA cycle and oxidative phosphorylation, forcing cells to shift toward the less efficient glycolytic pathway. Although this metabolic reprogramming acts as a stress response to energy shortage, it further reduces energy utilization efficiency and promotes the accumulation of harmful metabolites [15]. More critically, during mitochondrial energy conversion, dysfunctional electron transport chains (ETC) become major sources of reactive oxygen species (ROS). Excessive ROS attacks biological macromolecules, exacerbating oxidative damage and forming a vicious cycle where energy metabolism disorders and oxidative damage mutually intensify. This ultimately triggers an irreversible senescence process [16,17]. Therefore, implementing effective postharvest energy regulation is crucial for maintaining the quality of *A. bisporus* and delaying its senescence.

Modulating energy metabolism through controlled atmospheres represents a key approach to curb accelerated postharvest metabolic rates in *A. bisporus* [18]. Currently, high-CO_2_-modified atmosphere packaging has been proven effective in enhancing postharvest storage stability and delaying senescence [19]. For example, Wu found that Nano-PM can effectively activate antioxidant enzymes, inhibit the accumulation of ROS, and significantly reduce oxidative damage to lipids and proteins by regulating the gas microenvironment of *A. bisporus* [20]. Consequently, it maintains a relatively high energy level and extends the shelf life of the mushroom. Similarly, Li demonstrated that an appropriate O_2_/CO_2_ ratio effectively delays postharvest senescence in *A. bisporus* by maintaining high ATP levels and energy charge, as well as regulating the activity and gene expression of succinate dehydrogenase (SDH) and cytochrome C oxidase (CCO) [21]. Furthermore, amino acid metabolism and purine metabolism collectively support and regulate cellular energy metabolism by supplying substrates and facilitating energy flow [22,23]. Previous studies using transcriptomics and proteomics have shown gene and protein changes in mitochondria of postharvest *A. bisporus* [24,25]. However, changes at these levels do not fully translate into metabolic function. Metabolites, as end products of biological processes, reflect physiological states more directly. Therefore, to fully understand energy metabolism regulation, metabolomics is needed; this helps identify actual changes in key metabolites.

To address these knowledge gaps and elucidate the molecular mechanisms by which elevated CO_2_-modified atmosphere packaging delays postharvest senescence in *A. bisporus*, this study builds upon previous findings that 6% CO_2_ enhances energy status [24,25]. By integrating untargeted and targeted metabolomic analyses, we aim to identify key metabolic pathways regulated by elevated CO_2_ treatment that modulate postharvest energy levels. This work is expected to provide a theoretical foundation for enhancing the postharvest quality control system of *A. bisporus*.

## 2. Materials and Methods

### 2.1. Sample Preparation

**Figure 1:**
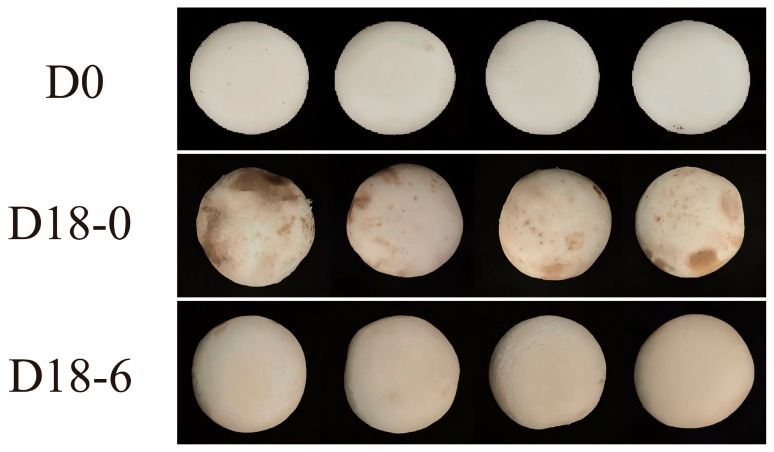
Appearance of *A. bisporus* after different postharvest treatments stored at 4 °C for 18 d.

The fresh *A. bisporus* needed for this experiment were purchased from a local farmers’ market (Jiangsu Yuguan Modern Agricultural Science and Technology Co., Ltd., Lianyungang, China). After harvest, they were transported to the laboratory under refrigeration. After pre-cooling at 4 °C for 24 h, those with uniform size and maturity, free from pests, umbrella opening, and mechanical damage were selected for subsequent experiments. In previous studies, the impact of elevated CO_2_ concentrations on the postharvest quality of double-packed *A. bisporus* was assessed by contrasting a gradient of CO_2_ levels (6%, 12%, 18%) against ambient atmospheric CO_2_ conditions. A suite of key postharvest quality parameters—including surface browning, relative electrical conductivity, textural characteristics, and cellular energy status—was continuously monitored throughout the storage period. The results demonstrated that a CO_2_ concentration of 6% exerted a significant preservative effect on the postharvest quality of *A. bisporus* [24,25]. On the basis of these observations, modified atmosphere packaging (MAP) with 6% CO_2_ was designated as the optimal treatment for the subsequent experiments in this study. The *A. bisporus* were randomly divided into two groups and placed in high-density polyethylene airtight boxes (200 × 140 × 65 mm). One group was filled with 6% CO_2_, 79% N_2_, and 15% O_2_, and the other group was the air treatment group, filled with 79% N_2_ and 21% O_2_. All samples were stored in a constant temperature and humidity chamber at 4 °C with a relative humidity of 90% for 18 d, which effectively reduced the respiratory rate of postharvest *A. bisporus*. To minimize individual variation, a pooled sampling strategy was employed. For each biological replicate, 3 whole mushrooms were randomly selected from the corresponding experimental group. These fruiting bodies were diced, pooled together, and immediately flash-frozen in liquid nitrogen before being ground into a homogenous powder. The powdered samples were then stored at −80 °C. For subsequent biochemical assays and metabolite extractions, specific amounts of this well-mixed frozen powder were accurately weighed according to each analytical protocol. The phenotype of *A. bisporus* under different treatments is shown in Figure 1.

Three experimental groups were established as follows: (1) D0—freshly harvested *A. bisporus* on day 0; (2) D18-0—*A. bisporus* stored for 18 d under ambient air conditions; (3) D18-6—*A. bisporus* stored for 18 d under 6% CO_2_ atmosphere. Three experimental groups were established as follows: (1) D0—freshly harvested *A. bisporus* on day 0; (2) D18-0—*A. bisporus* stored for 18 d under ambient air conditions; (3) D18-6—*A. bisporus* stored for 18 d under 6% CO_2_ atmosphere. 

### 2.2. UHPLC-OE-MS Untargeted Metabolomics Assay

The samples (25 mg) were taken and lyophilized, and mixed with beads and 1000 μL of extraction solution (MeOH:ACN:H_2_O, 2:2:1 (*v*/*v*)). The extraction solution contains deuterated internal standards. The mixed solution was vortexed for 30 s. Then, the mixed samples were homogenized (35 Hz, 4 min) and sonicated for 5 min in a 4 °C water bath; this step was repeated three times. The samples were incubated for 1 h at −40 °C to precipitate proteins. Then, the samples were centrifuged at 8000× *g* for 15 min at 4 °C, and the supernatant was transferred to a fresh glass vial for analysis.

The Vanquish (Thermo Fisher Scientific, Waltham, MA, USA) ultra-high-performance liquid chromatograph was used for chromatographic separation of the target compounds through a Phenomenex Kinetex C18 (2.1 mm × 50 mm, 2.6 μm) liquid chromatography column. Mobile phase A was an aqueous solution containing 0.01% acetic acid; mobile phase B was a mixture of isopropanol (IPA) and acetonitrile (ACN) (volume ratio 1:1). The temperature of the autosampler was set to 4 °C, and the injection volume was 2 μL.

The parameter settings of the electrospray ionization (ESI) source were as follows: sheath gas flow rate 50 Arb, auxiliary gas (Aux gas) flow rate 15 Arb, capillary temperature 320 °C; full-scan primary mass spectrometry resolution 60,000, secondary mass spectrometry resolution 15,000; collision energy was normalized collision energy (SNCE) 20/30/40; the spray voltage was 3.8 kV in positive ion mode and −3.4 kV in negative ion mode.

### 2.3. UHPLC-MRM-MS Targeted Metabolomics Assay

We weighed a 25 mg sample into a 2 mL EP tube. A total of 500 μL extraction agent (MeOH:H_2_O = 3:1, containing internal standards) was added to the sample, and two 3.2 mm beads were added to the mixture. Samples were homogenized at 40 Hz for 4 min, and were sonicated in an ice-water bath for 5 min. The homogenization and sonication cycle was repeated three times. After that, samples were incubated at −40 °C for 1 h. Then, all the samples were centrifuged at 8000× *g* and 4 °C for 15 min. A total of 400 μL of supernatant was transferred to a 2 mL EP tube and dried under vacuum. The residue was reconstituted in 200 μL of methanol/acetonitrile/water (1:1:2, *v*/*v*/*v*), filtered, and the liquid was transferred to an autosampler vial for analysis.

An Agilent 1290 Infinity II ultra-high-performance liquid chromatograph (Santa Clara, CA, USA) was used for chromatographic separation of the target compounds via an Atlantis Premier BEH Z-HILIC Column (Milford, MA, USA) (1.7 µm, 2.1 mm × 150 mm). The mobile phase A for liquid chromatography was ultrapure water/acetonitrile = 10:90, containing 10 mmol L^−1^ ammonium acetate; mobile phase B was ultrapure water/acetonitrile = 90:10, containing 10 mmol L^−1^ ammonium acetate. The sample tray temperature was 6 °C, and the injection volume was 2 μL.

Mass spectrometry data were acquired in multiple reaction monitoring (MRM) mode using a SCIEX Triple Quad 6500+ triple quadrupole mass spectrometer (Framingham, MA, USA) equipped with an IonDrive Turbo V ESI ion source (Framingham, MA, USA). Ion source parameters were set as follows: Curtain Gas = 35 psi, IonSpray Voltage = +5500 V/−4500 V, Temperature = 400 °C, Ion Source Gas 1 = 50 psi, and Ion Source Gas 2 = 50 psi.

### 2.4. Metabolomics Data Processing

In this study, both untargeted and targeted metabolomics experiments were set up with 3 biological replicates. Principal component analysis (PCA) and orthogonal partial least squares discriminant analysis (OPLS-DA) were performed using SIMCA 14.1 software. The screening criteria for differential metabolites were as follows: variable importance in projection (VIP) value derived from OPLS-DA > 1, and a *p*-value derived from a *t*-test < 0.05. The heatmap of hierarchical cluster analysis was plotted using the MetWare Cloud Platform (https://cloud.metware.cn/, accessed on 28 May 2026); the Kyoto Encyclopedia of Genes and Genomes (KEGG) enrichment analysis and pathway mapping of differential metabolites were completed using the MetaboAnalyst 5.0 online tool (https://www.metaboanalyst.ca/, accessed on 28 May 2026) and plotted using the Bioinformatics online platform (https://www.bioinformatics.com.cn/, accessed on 28 May 2026).

### 2.5. Measurement of the Activity of Key Rate-Limiting Enzymes in Related Metabolic Pathways

The activity of phosphofructokinase (PFK) was determined using a PFK activity assay kit (Beijing Solarbio Science & Technology Co., Ltd., Beijing, China). One unit of enzyme activity was defined as the consumption of 1 nmol of NADH converted to 1 nmol of NAD^+^ per minute per gram of tissue in the reaction system, with results expressed in U g^−1^ fresh weight.

The activity of hexokinase (HK) was measured using a HK activity assay kit (Beijing Solarbio Science & Technology Co., Ltd., Beijing, China). One unit of enzyme activity was defined as the production of 1 nmol of NADPH per minute per gram of tissue, with results expressed in U g^−1^ fresh weight.

The activity of pyruvate kinase (PK) was assayed using a PK activity assay kit (Beijing Solarbio Science & Technology Co., Ltd., Beijing, China). One unit of enzyme activity was defined as the consumption of 1 nmol of NADH per minute per gram of tissue, with results expressed in U g^−1^ fresh weight.

The activity of succinate dehydrogenase (SDH) was measured using an SDH activity assay kit (Beijing Solarbio Science & Technology Co., Ltd., Beijing, China). One unit of enzyme activity was defined as the consumption of 1 nmol of 2,6-dichlorophenol indophenol per minute per gram of tissue in the reaction system, with results expressed in U g^−1^ fresh weight.

The activity of NAD-malate dehydrogenase (NAD-MDH) was determined using the NAD-MDH Activity Assay Kit (Beijing Solarbio Science & Technology Co., Ltd., Beijing, China). The enzyme activity unit is defined as follows: per gram of tissue consumes 1 nmol of NADH per minute in the reaction system, and the results are expressed as U g^−1^ fresh weight.

The activity of fumarase (FUM) was determined using a FUM activity assay kit (Shanghai Beyotime Biotechnology Co., Ltd., Shanghai, China). One unit of enzyme activity was defined as the production of 1 μmol of NADPH per minute at 37 °C, with results expressed in U g^−1^ fresh weight.

The activity of glutamine synthetase (GS) was determined using a GS activity assay kit (Beijing Solarbio Science & Technology Co., Ltd., Beijing, China). One unit of enzyme activity was defined as a change in absorbance of 0.005 at 540 nm per minute per gram of tissue in the reaction system, with results expressed in U g^−1^ fresh weight.

The activity of xanthine oxidase (XOD) was determined using the XOD Activity Assay Kit (Beijing Solarbio Science & Technology Co., Ltd., Beijing, China). The enzyme activity unit is defined as follows: per gram of tissue catalyzes the production of 1 nmol of NO_2_^−^ per minute, and the results are expressed as nmol h^−1^ g^−1^ fresh weight.

### 2.6. Real-Time Fluorescence Gene Expression Assay

The RNA of *A. bisporus* was extracted using the Vazyme Fungal RNA Kit (Nanjing Vazyme Biotech Co., Ltd., Nanjing, China). The concentration and quality of the extracted RNA were detected with an ultra-micro spectrophotometer, with a sample loading volume of 2 μL. RNA was reverse-transcribed into cDNA using HiScript III RT SuperMix (Nanjing Vazyme Biotech Co., Ltd., Nanjing, China) for qPCR (+gDNA Wiper) for later use. The gene expression level was detected according to the instructions of ChamQ Blue Universal SYBR qPCR Master Mix (Nanjing Vazyme Biotech Co., Ltd., Nanjing, China), and the relative expression level of the target gene mRNA was calculated using the 2^−ΔΔCt^ formula. The internal reference gene was GADPH. Primers were designed on NCBI and synthesized by Sangon Biotech (Shanghai) Co., Ltd. (Shanghai, China). The specific primer sequences are listed in Table A1.

### 2.7. Statistical Analysis

All data are expressed as the mean ± standard deviation (SD) of three independent biological replicates (*n* = 3). One-way analysis of variance (one-way ANOVA) and Duncan’s multiple comparison test were performed using SPSS 21.0 software to compare the differences between the 6% CO_2_-modified atmosphere packaging treatment group and the air treatment group, with the significance level set at *p* < 0.05. The charts in this paper were drawn using Origin 2018 and Graphpad Prism 10.1.2 software.

## 3. Results

### 3.1. Analyses of PCA and OPLS-DA

**Figure 2:**
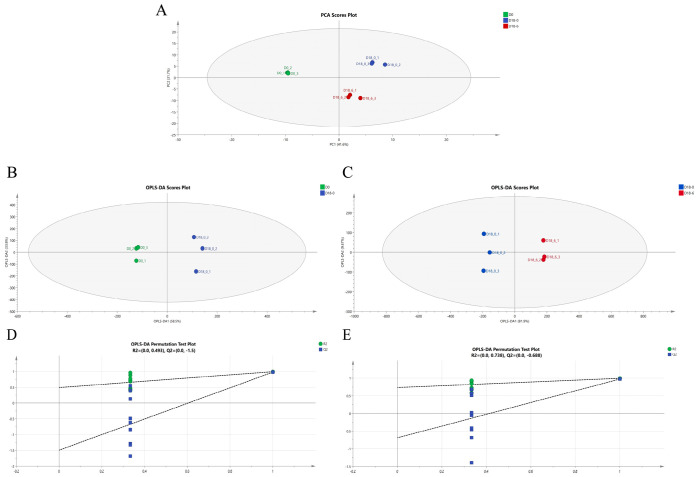
Non-targeted metabolomics: Principal component analysis (**A**); OPLS-DA D0 vs. D18-0 (**B**); D18-0 vs. D18-6 (**C**); OPLS-DA. Permutation testing: D0 vs. D18-0 (**D**) and D18-0 vs. D18-6 (**E**).

To gain a comprehensive understanding of the metabolomic dynamics of *A. bisporus*, an untargeted metabolomics approach based on UHPLC-OE-MS was employed to systematically identify and quantify all metabolites present in *A. bisporus*, including primary and secondary metabolites, and its PCA score plots for D0, D18-0, and D18-6 were also established (Figure 2A). The PCA is used to observe the overall distribution of the sample. R2X (cum) represents the interpretable rate of the model, and R2X >  0.4 is generally considered better [26]. The R2X in the PCA score plot was 0.732 (Table A2). The three biological replicates in each group were clustered tightly together, indicating that the experiment was stable and reproducible. In the PC1-PC2 plane, the cumulative explained variance was 73%, and the three groups of samples exhibited a gradient spatial distribution. At the same time, D18-0 and D18-6 were obviously separated, indicating that 6% CO_2_ treatment significantly influenced the metabolite profile of *A. bisporus* during storage.

The OPLS-DA model was further applied to extend the regression capabilities of PCA for visualizing inter-group differences in postharvest *A. bisporus* (D0 vs. D18-0 and D18-0 vs. D18-6) (Figure 2B–E). R2Y (cum) represents the cumulative proportion of variance in the response variable Y explained by the model, while Q2 (cum) reflects the predictive ability of each model. In the D0 vs. D18-0 model, the OPLS-DA model describes 92.3% of the variance in X (R2X = 0.923) and 99.2% of the variance in response Y (R2Y = 0.992), and Q2  =  0.979. In the D18-0 vs. D18-6 model, the OPLS-DA model describes 91.6% of the variance in X (R2X = 0.916) and 99.5% of the variance in response Y (R2Y = 0.99.5), and Q2  =  0.975. These results demonstrate that both models are stable and exhibit excellent predictive characteristics [27,28].

### 3.2. Screening and Analysis of Differential Metabolites

**Figure 3:**
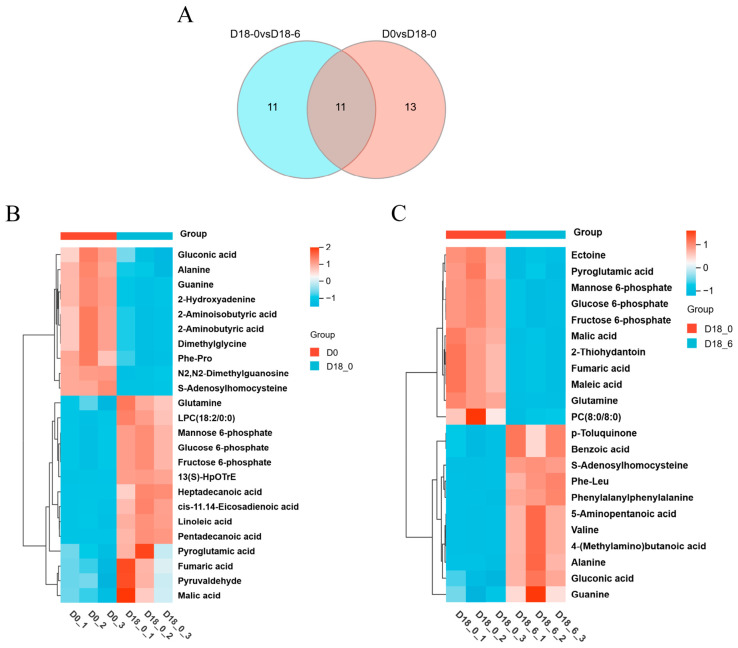
Venn diagram (**A**). Heatmap of differential metabolite clustering: D0 vs. D18-0 (**B**), and D18-0 vs. D18-6 (**C**). The horizontal coordinate is the sample name, and the vertical coordinate is the differential metabolite. The color in the heatmap indicates the normalized value of the relative content of the differential metabolites, with red representing high content and blue representing low content.

In the OPLS-DA model, scatter plots of variable importance in projection (VIP) were used to score metabolites more relevant to each group in the dataset [29]. Therefore, differential metabolites associated with postharvest quality changes in *A. bisporus* were subsequently screened using the following criteria: VIP ≥ 1, univariate statistical analysis (*p* < 0.05), and |log_2_FC| > 1. A Venn diagram effectively illustrated both distinct and shared differential metabolites among the groups (Figure 3A). In the D0 vs. D18-0 and D18-0 vs. D18-6 comparisons, twenty-four and twenty-two differential metabolites were identified, respectively. Eleven differential metabolites were common to both comparisons, including Glucose-6-phosphate (G-6-P), Fructose-6-phosphate (F-6-P), Mannose-6-phosphate (M-6-P), Fumarate (Fum), Glutamine (Gln), Alanine (Ala), Guanine (Gua), Pyroglutamate (pGlu), Malate (Mal), Gluconate (Gnt), and S-Adenosylhomocysteine (SAH). The differences in the relative content of these substances are thought to be the result of differences in CO_2_ concentration treatments.

The clustering analysis revealed substantial metabolic differences between D0 vs. D18-0 and D18-0 vs. D18-6. As shown in Figure 3B,C (Table A3 and Table A4), in the D0 vs. D18-0 comparison, prolonged storage resulted in the upregulation of fourteen compounds and downregulation of ten compounds in *A. bisporus*. Among these, G-6-P, F-6-P, M-6-P, Fum, Gln, pGlu, and Mal were significantly upregulated. In contrast, Ala, Gua, and Gnt were significantly downregulated. In the D18-0 vs. D18-6 comparison, 6% CO_2_ treatment led to an upregulation of eleven compounds and a downregulation of eleven compounds. Notably, Ala, Gua, Gnt, and SAH became significantly upregulated, while phosphorylated sugars, Fum, Gln, pGlu, and Mal were significantly downregulated. These changed substances are important for the treatment to delay the senescence of postharvest *A. bisporus* and maintain its energy metabolism. These metabolites collectively form the core network of energy metabolism in *A. bisporus*, and their dynamic changes directly reflect the adjustment and remodeling of the organism’s energy metabolism state under different CO_2_ treatment conditions.

### 3.3. Analysis of KEGG Pathway

**Figure 4:**
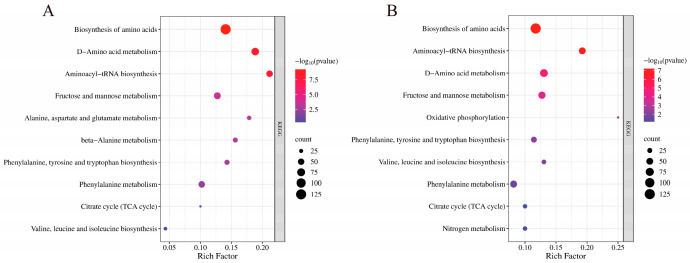
KEGG analysis: D0 vs. D18-0 (**A**) and D18-0 vs. D18-6 (**B**). The size of the circle indicates the number of differentially expressed metabolites of the mapped pathway; the larger the circle, the higher the number. The color of the circle indicates the size of the *p*-value; the redder the color, the smaller the *p*-value.

KEGG enrichment analysis was performed on the differential metabolites of the screened *A. bisporus* during postharvest storage. Listed are the ten metabolic pathways with high significance in the D0 vs. D18-0 group and D18-0 vs. D18-6 group, respectively (Figure 4A,B). In the D0 vs. D18-0 comparison, pathways that included cofactor biosynthesis, amino acid biosynthesis, nucleotide metabolism, purine metabolism, 2-oxocarboxylic acid metabolism, glyoxylate and dicarboxylate metabolism, butanoate metabolism, the TCA cycle, and glycolysis/gluconeogenesis were significantly enriched. In the D18-0 vs. D18-6 comparison, significant enrichment was observed in cofactor biosynthesis, amino acid biosynthesis, carbon metabolism, purine metabolism, the pentose phosphate pathway, glyoxylate and dicarboxylate metabolism, alanine, aspartate and glutamate metabolism, the TCA cycle, one-carbon pool by folate, and glycolysis/gluconeogenesis. Integrating the enrichment results with the previously mentioned changes in metabolites clearly highlights four common pathways: amino acid biosynthesis, purine metabolism, the TCA cycle, and glycolysis/gluconeogenesis as the core factors influencing the postharvest energy status of *A. bisporus*. Among these, glycolysis/gluconeogenesis and the TCA cycle, as the central hubs of energy metabolism, are responsible for the production of basic energy; purine metabolism directly governs the synthesis and recycling of ATP, and amino acid biosynthesis exerts a profound impact on the balance and flux of the entire energy network in *A. bisporus* through its close interaction with central carbon metabolism.

### 3.4. Quantitative Results of Differential Metabolites

**Figure 5:**
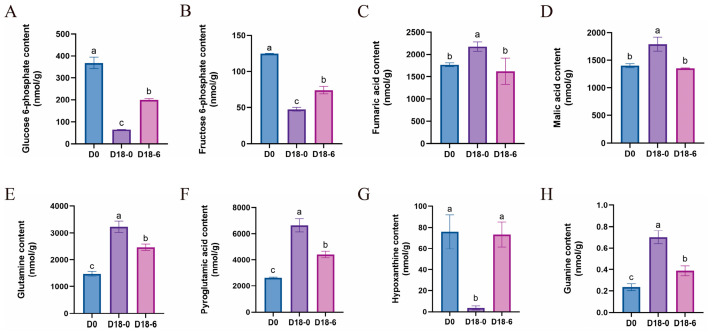
Comparison chart of multi-index metabolite contents. (**A**) Glucose 6-phosphate; (**B**) Fructose 6-phosphate; (**C**) Fumaric acid; (**D**) Malic acid; (**E**) Glutamine; (**F**) Pyroglutamic acid; (**G**) Hypoxanthine; (**H**) Guanine. Data are presented as mean ± SD (*n* = 3). Different lowercase letters indicate significant differences (*p* < 0.05) between treatment groups within the same storage period.

In the study on postharvest metabolism of *A. bisporus*, based on the previous untargeted metabolomics results, we focused on differential metabolites and pathways related to energy metabolism. Absolute quantification of key energy metabolites was performed using targeted metabolomics. As shown in Figure 5A,B, the glycolysis-related phosphorylated sugars G-6-P and F-6-P shared similar variation patterns. Their contents declined in the D18-0 group relative to D0, demonstrating that glycolysis was suppressed during postharvest storage of *A. bisporus*. By comparison, the accumulation of these two sugars was markedly enhanced in the D18-6 group compared with D18-0, revealing that elevated CO_2_ could promote glycolytic metabolism and thereby facilitate energy maintenance in *A. bisporus*. Figure 5C,D present the quantitative results for Fum and Mal. As intermediate products of the TCA cycle, both showed significant increases in the D18-0 group. Their subsequent return to near D0 levels in the D18-6 group suggests that elevated CO_2_ treatment helped maintain normal TCA cycle operation in *A. bisporus*, and alleviated abnormal accumulation of cycle intermediates. The contents of Gln and pGlu, shown in Figure 5E,F, exhibited extremely significant accumulation in the D18-0 group. Their concentrations were substantially higher than in both D0 and D18-6 groups. This indicates that the air storage environment strongly promoted protein degradation or disruption in related amino acid metabolic pathways. Figure 5G,H display the content changes for Hx and Gua, which showed opposing trends. Hx levels decreased to a minimum in the D18-0 group, while Gua content rose significantly to its highest point. This inverse pattern suggests that the catabolism of purine nucleotides responds differently to storage stress across distinct branch pathways.

In summary, these targeted quantification results demonstrate that 6% CO_2_ treatment effectively maintained postharvest metabolic homeostasis in *A. bisporus*. This was achieved by regulating core energy metabolism pathways and mitigating oxidative damage, thereby delaying the senescence process.

### 3.5. Detection of Key Enzyme Activities

**Figure 6:**
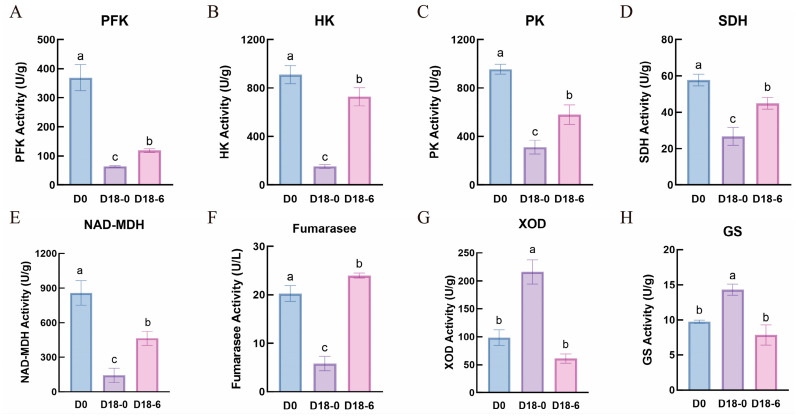
Activity of key enzymes in metabolic pathways. (**A**) PFK; (**B**) HK; (**C**) PK; (**D**) SDH; (**E**) NAD-MDH; (**F**) Fumarase; (**G**) XOD; (**H**) GS. Data are presented as mean ± SD (*n* = 3). Different lowercase letters indicate significant differences (*p* < 0.05) between treatment groups within the same storage period.

PFK, HK, and PK are key rate-limiting enzymes in the glycolytic pathway. They directly regulate the efficiency of glucose catabolism and ATP supply, playing a central role in postharvest energy metabolism and the senescence of *A. bisporus* [30]. As shown in Figure 6A–C, PFK, HK, and PK activities significantly decreased in the D18-0 group compared to D0. This indicates a general decline in the glycolytic pathway during natural senescence, leading to impaired energy metabolism. The 6% CO_2_ treatment effectively reversed this trend. Enzyme activities in the D18-6 group were significantly higher than in D18-0. This demonstrated that CO_2_ treatment maintained key glycolytic enzyme activities, thereby delaying the decline of postharvest energy metabolism in *A. bisporus*.

Figure 6D–F present activity changes for SDH, NAD-MDH, and FUM. All three enzymes are key enzymes in the TCA cycle and affect the postharvest energy metabolism of *A. bisporus*. After 18 d of air storage, SDH activity was strongly inhibited. This disruption directly impaired the coupling between the TCA cycle and the ETC, causing a sharp decline in cellular energy synthesis [31]. Simultaneously, internal TCA cycle flux was obstructed. Reduced FUM activity hindered the conversion of Fum to Mal, leading to Fum accumulation. The significant decrease in NAD-MDH activity indicated insufficient regeneration of oxaloacetate. This may result from feedback inhibition by high-energy metabolites (NADH/ATP). Collectively, these disruptions slowed or halted TCA cycle flux [32,33]. In contrast, 6% CO_2_ storage showed remarkable preservation effects. It successfully maintained SDH, FUM, and NAD-MDH activities at normal levels. This ensured the smooth operation of the TCA cycle and the stable supply of energy, as well as maintained all metabolic processes of *A. bisporus* in a steady state, and ultimately, effectively delayed the senescence process.

XOD is a key enzyme in purine ROS nucleotide catabolism. It catalyzes uric acid production and generates ROS [34]. As shown in Figure 6G, XOD activity peaked in D18-0. This clearly indicates that air storage strongly activated the purine catabolic metabolism in *A. bisporus*. Activity in D18-6 returned to D0 levels, showing that 6% CO_2_ storage effectively suppressed XOD activation. This maintained the hypoxanthine content close to fresh levels, demonstrating that 6% CO_2_ significantly preserves quality by delaying nucleotide degradation. GS is a key enzyme in glutamate degradation. As shown in Figure 6H, GS activity increased in D18-0 but decreased to D0 levels in D18-6. This indicates that 6% CO_2_ treatment inhibits GS, redirecting metabolic flux toward the TCA cycle to enhance energy production and delay postharvest senescence. Therefore, 6% CO_2_ treatment delays the postharvest senescence process of *A. bisporus* effectively by maintaining energy metabolism enzyme activities through multiple pathways, thereby ensuring a stable energy supply and slowing down metabolic decline.

### 3.6. qRT-PCR Validation

**Figure 7:**
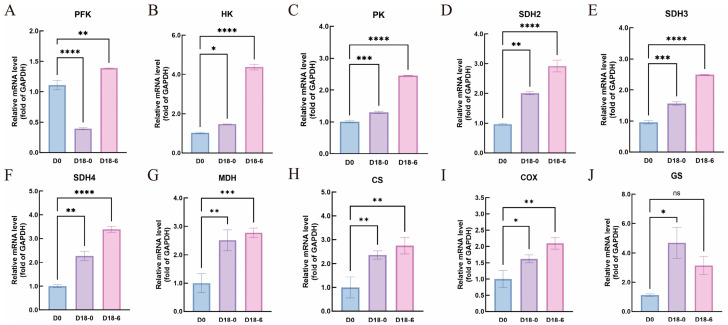
qRT-PCR validation analysis of the relative expression levels. (**A**) PFK; (**B**) HK; (**C**) PK; (**D**) SDH2; (**E**) SDH3; (**F**) SDH4; (**G**) MDH; (**H**) CS; (**I**) COX; (**J**) GS. *  *p*  <  0.05, ** *p*  <  0.01, *** *p * <  0.001, and **** *p*  <  0.0001; ns stands for no significant difference.

To validate the reliability of the metabolomics data of *A. bisporus*, ten key genes involved in glycolysis, the TCA cycle, and amino acid metabolism were selected for qRT-PCR verification. As shown in Figure 7, the qRT-PCR results and metabolomic as well as enzyme activity data mutually corroborated one another, confirming the reliability of the metabolomics findings and providing solid genetic evidence for the subsequent mechanistic interpretation. Specifically, the differential upregulation of key glycolytic genes (*HK*, *PFK*, *PK*) (Figure 7A–C) collectively indicates that 6% CO_2_ treatment effectively activated the glycolytic flux in postharvest *A. bisporus*. The sustained upregulation of *HK* ensured glucose phosphorylation and retention, supplying substrates for downstream metabolism. The marked enhancement of *PK* in D18-6 directly promoted substantial Pyr production, which not only provided a core entry point for the TCA cycle but also laid a solid foundation for maintaining cellular energy charge. The coordinated upregulation of TCA cycle key genes (*CS*, *SDH2*, *SDH3*, *SDH4*, and *MDH*) provides direct evidence that 6% CO_2_ treatment enhanced the postharvest cellular energy level of *A. bisporus* (Figure 7D–H). Upregulated *CS* implies more efficient entry of acetyl-CoA into the cycle. Enhanced expression of *SDH2*, *SDH3*, *SDH4*, and *MDH*, key catalytic and NADH-producing nodes, collectively drove abundant NADH/FADH_2_ synthesis. This greatly promoted the operation of the TCA cycle and mitochondrial ETC, enhancing oxidative phosphorylation and ultimately increasing ATP production [35]. Maintaining such energy levels is crucial for delaying energy-depletion senescence in postharvest *A. bisporus*. Significant upregulation of *COX* indicates enhanced terminal oxidative activity in mitochondria (Figure 7I). This ensures smooth electron flow through the chain and reduces excess ROS generation caused by electron leakage by mediating the dynamic equilibrium of the ETC and protecting mitochondrial structure and function [36]. The staged upregulation of *GS* suggests that 6% CO_2_ treatment helps maintain cellular osmotic balance and stabilizes amino acid metabolism (Figure 7J). Together, these results illustrate the regulatory mechanism through which elevated CO_2_ treatment enhances the energy status and delays senescence in postharvest *A. bisporus*.

## 4. Discussion

**Figure 8:**
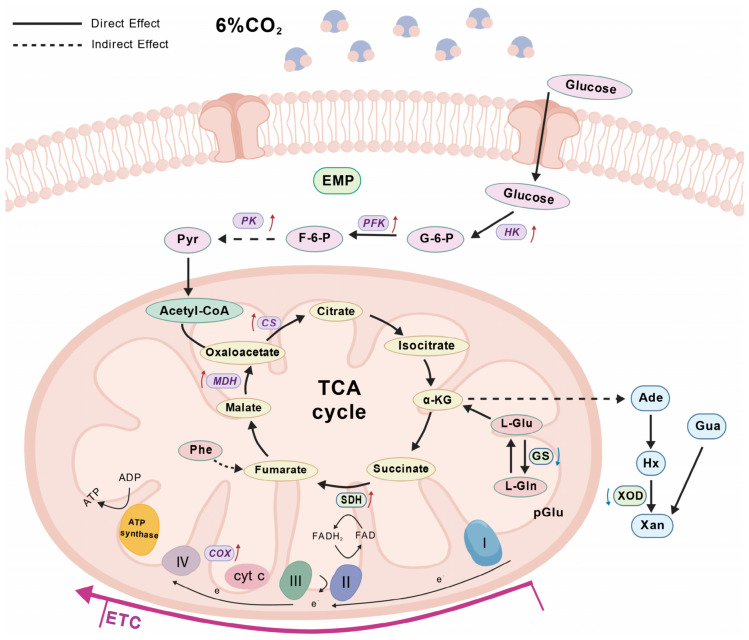
Schematic diagram of the mechanism by which 6% CO_2_ treatment delays postharvest senescence of *A. bisporus* through modulating the energy metabolism network. The red upward arrows represent upregulation, and the green downward arrows represent downregulation.

Postharvest senescence in *A. bisporus* is a complex biological process, in which the rapid decline of energy metabolism serves as a key factor contributing to the deterioration of postharvest quality [37]. In our previous research, we found that 6% CO_2_ treatment enhances the activity of the mitochondrial respiratory chain, increases ATP content and energy charge (EC), and improves the postharvest energy status of *A. bisporus*, thereby elevating its quality [24]. In this study, through untargeted and targeted metabolomics analyses, we further clarified the key pathways and molecular mechanisms by which elevated CO_2_-modified atmosphere packaging regulates postharvest energy metabolism. The results showed that elevated CO_2_ treatment delays postharvest senescence by promoting glycolysis, enhancing the core driving force and integrity of the TCA cycle, and inhibiting the abnormal catabolism of macromolecules such as amino acids and purine nucleotides induced by an energy crisis (Figure 8).

### 4.1. Elevated CO_2_ Treatment Promotes Postharvest Glycolysis

Glycolysis is the initial step of postharvest energy metabolism in *A. bisporus*. By efficiently decomposing endogenous carbohydrates, it directly generates basic energy (ATP) and metabolic substrates required for life activities, thereby providing sufficient energy support for key physiological needs such as the maintenance of postharvest vital activities, repair of cellular structures, and delay of quality deterioration [38].

Our research findings revealed that, compared with the D18-0 group, the 6% CO_2_ treatment significantly increased the activities of HK, PFK, and PK. It also upregulated their gene expression and promoted the accumulation of G-6-P, F-6-P, and Pyr in postharvest *A. bisporus*. Previous studies have shown that elevated CO_2_ environments can regulate the expression of Hxts, promote glucose transport, and participate in the glycolytic process [39]. Meanwhile, as the initiating enzyme, HK, upon sensing relatively ample glucose supply, strengthens the glucose phosphorylation process, providing sufficient substrate for the subsequent glycolytic process [40]. Furthermore, PFK, the most critical rate-limiting enzyme and primary regulatory site in glycolysis, is strictly controlled by cellular energy status. CO_2_ treatment alleviated ATP’s allosteric inhibition of PFK, thereby accelerating the overall glycolytic flux [41]. Finally, the activation of PK at the pathway terminus ensured the efficient conversion of phosphoenolpyruvate to Pyr, accompanied by net ATP generation, providing direct energy support [42]. Our results confirmed that elevated CO_2_ treatment accelerates postharvest glycolysis in *A. bisporus* by maintaining high activities of HK, PFK, and PK. This not only provides immediate ATP for the postharvest vital activities of *A. bisporus* but also supplies the essential Pyr substrate for the continuous operation of the subsequent TCA cycle, laying an energy foundation for delaying the postharvest senescence of *A. bisporus* at the source. This result is consistent with the findings of Wang et al. [43].

### 4.2. Elevated CO_2_ Treatment Enhances Postharvest TCA Cycle Efficiency

The TCA cycle is the core hub of postharvest energy metabolism in *A. bisporus*. It not only converts Pyr produced by glycolysis into acetyl-CoA but also generates large amounts of reducing power (NADH and FADH_2_) through a series of dehydrogenation reactions. This reducing power drives the ETC to perform oxidative phosphorylation, enabling efficient ATP synthesis. This tightly coupled process provides fundamental energy support for maintaining the postharvest quality of *A. bisporus* [15].

**Figure A1:**
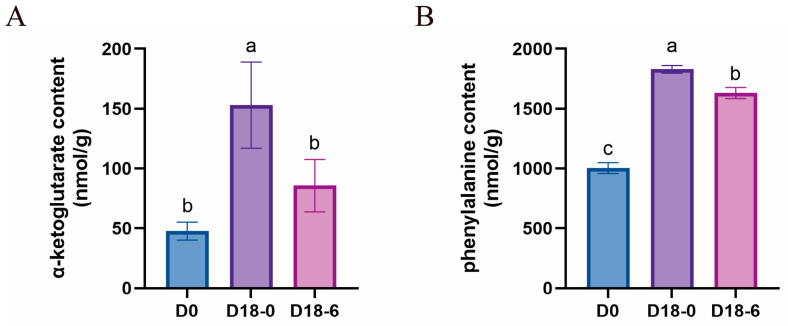
Comparison chart of multi-index metabolite contents. (**A**) α-ketoglutarate content; (**B**) phenylalanine content. Data are presented as mean ± SD (*n* = 3). Different lowercase letters indicate significant differences (*p* < 0.05) between treatment groups within the same storage period.

Our metabolomic results showed that, compared with the D18-0 group, elevated CO_2_ treatment significantly downregulated differential metabolites in the TCA cycle, for example, α-ketoglutarate (α-KG) (Figure A1A). Meanwhile, by increasing the activities of *MDH* and *FUM*, and upregulating the expression of MDH and FUM, the treatment reduced the accumulation of metabolites, including Fum and Mal. Previous studies have indicated that elevated CO_2_ can enhance the efficiency of oxidative decarboxylation of Pyr (the end product of glycolysis) in postharvest edible fungi, promoting the accumulation of acetyl-CoA [24,44]. This provides sufficient substrates for the TCA cycle, thereby accelerating its operation, reducing the abnormal accumulation of TCA products, and supplying more reducing power for the subsequent ETC [45]. As previously reported, elevated CO_2_ treatment can activate the Ca^2+^ signaling pathway, specifically upregulating the expression of FUM and MDH [46]. Based on this finding, we hypothesize that the enhanced activities of FUM and MDH observed in our current study may be driven by this upstream Ca^2+^-dependent regulatory cascade [46]. This promotes the conversion of Fum–Mal–oxaloacetate (OAA), enhancing the efficiency of carbon skeleton flow and energy supply. Our study demonstrated that elevated CO_2_ treatment drives the overall enhancement of metabolic flux from Pyr conversion and acetyl-CoA production to TCA cycle operation, and coordinately upregulates the activity and expression of FUM and MDH. This establishes an efficient energy metabolism network, providing fundamental support for maintaining the postharvest quality of *A. bisporus*. This finding is consistent with the conclusions of Tong et al. [25].

We also found that elevated CO_2_ treatment significantly upregulated the expression levels of genes encoding SDH (i.e., *SDH2*, *SDH3*, and *SDH4*) and CCO (*COX*). SDH is the only enzyme complex in the TCA cycle embedded in the inner mitochondrial membrane. As Complex II of the ETC, it directly transfers electrons obtained via FADH_2_ to the ubiquinone pool, promoting ATP synthesis [47]. Meanwhile, CCO, as the terminal enzyme of the ETC (Complex IV), is responsible for transferring electrons from cytochrome c to oxygen (ultimately generating water) and pumping protons—this is a key driving step for establishing the proton gradient [48]. Previous studies have confirmed that CO_2_ treatment can regulate genes related to mitochondrial biogenesis, promoting the expression of core oxidative phosphorylation components, including SDH and CCO [49]. The coordinated upregulation of SDH and CCO greatly alleviates electron congestion in the ETC, reduces electron leakage and ROS production, and drives more efficient oxidative phosphorylation for ATP synthesis. This provides solid energy support for stabilizing the postharvest quality of *A. bisporus*, which is consistent with the conclusions of Zan et al. [50].

### 4.3. Elevated CO_2_ Treatment Coordinates Processes of Amino Acid and Purine Metabolism

Amino acid metabolism is an important energy supply pathway for postharvest *A. bisporus*. By degrading free amino acids, it directly provides key carbon skeleton intermediates such as α-KG for the TCA cycle, thereby injecting stronger metabolic driving force into the TCA cycle [23]. Purine metabolism, on the other hand, regulates the decomposition of ATP/ADP/AMP, slowing down the senescence physiological process of postharvest edible fungi caused by energy decline [51].

Our research results showed that in terms of amino acid metabolism, compared with the D18-0 group, elevated CO_2_ treatment significantly reduced the activity of GS in *A. bisporus*, inhibited the synthesis of Gln and pGlu, and decreased the content of phenylalanine (Phe) (Figure A1B). Studies have found that elevated CO_2_ treatment reduces energy consumption by inhibiting protein degradation, leading to a decrease in GS activity. This further promotes the rebalancing of amino acid metabolism and ultimately consolidates and improves the postharvest energy status of *A. bisporus* by enhancing respiration [52,53]. In addition, CO_2_ treatment promotes the degradation of exogenous free amino acids such as Gln and Phe. These amino acids are converted into key intermediates such as α-KG and Fum through transamination, which directly replenish the TCA cycle and drive the continuous production of ATP [17].

In terms of purine metabolism, compared with the D18-0 group, elevated CO_2_ treatment significantly reduced the postharvest Gua content of *A. bisporus*, promoted the accumulation of Hx, and significantly weakened the activity of XOD. This is because elevated CO_2_ treatment promotes the accumulation of Hx precursor substances such as adenine (Ade), leading to blocked downstream conversion of Hx and its subsequent accumulation. This change effectively blocks the massive production of ROS catalyzed by XOD, reduces the net consumption of nucleotides (AMP/GMP/IMP), and improves the overall energy efficiency of *A. bisporus* [54,55].

Our results confirmed that elevated CO_2_ treatment coordinates the processes of amino acid metabolism and purine metabolism by establishing energy homeostasis, blocking oxidative damage pathways, and thereby comprehensively improving the energy efficiency and cellular integrity of postharvest *A. bisporus*. This conclusion is mutually corroborated by the findings of Shang et al. [24].

Upstream of these metabolic responses, our prior work revealed that elevated CO_2_ actively stimulates the Hog1-MAPK signaling pathway, significantly upregulating the core sensor kinase genes *Hog1* and *Pbs2*. This primary signaling event acts to buffer oxidative stress, downregulate cell cycle-related proteins, and bolster cellular energy reserves [25]. Building upon this, we propose that the profound metabolic restructuring characterized in the present study—specifically, the amplified glycolytic flux, accelerated TCA cycle turnover, and the energy-conserving attenuation of amino acid metabolism and purine metabolism—functions as the direct biochemical effector network of the Hog1-MAPK/Pbs2 cascade. Collectively, this integrated “sensor-to-metabolism” axis drives the metabolic adaptations that effectively delay senescence and maintain the quality of *A. bisporus*.

## 5. Conclusions

In this study, we systematically revealed the unique regulatory mechanism by which 6% elevated CO_2_ modulates energy metabolism in postharvest *A. bisporus*. Different from conventional preservation approaches, elevated CO_2_ treatment exerted a distinctive metabolic regulation pattern: it significantly induced the expression of core rate-limiting enzymes (HK, PFK, and PK), thereby empowering glycolysis and providing sufficient intermediate substrates for the subsequent TCA cycle. Further analysis demonstrated that critical TCA cycle enzymes (SDH, MDH, and FUM) were synchronously activated, which greatly optimized TCA cycle operation efficiency. Meanwhile, the coordinated upregulation of ETC-related genes further boosted ATP biosynthesis and ensured cellular energy supply. Notably, elevated CO_2_ orchestrated the crosstalk of amino acid and purine metabolism to reinforce TCA cycle flux and effectively alleviate ROS-mediated oxidative injury. Collectively, these multi-pathway synergistic responses constructed a fine-tuned energy regulatory network, which successfully stabilized metabolic homeostasis and ultimately retarded postharvest senescence of *A. bisporus*. This work uncovers a novel CO_2_-mediated energy regulation cascade in edible fungi and provides an innovative theoretical basis for improving postharvest storage quality via controlled atmosphere regulation. Nevertheless, our previous studies have identified the Hog1-MAPK cascade as a primary upstream responder to CO_2_ signals. Furthermore, the precise molecular crosstalk between these initiating kinase cascades and the specific metabolic enzymes elucidated in this study remains to be fully mapped and requires further validation in future investigations.

## Data Availability

The original contributions presented in this study are included in the article. Further inquiries can be directed to the corresponding author.

## Abbreviations

The following abbreviations are used in this manuscript:

HK	Hexokinase
PFK	Phosphofructokinase
PK	Pyruvate Kinase
G-6-P	Glucose-6-phosphate
F-6-P	Fructose-6-phosphate
M-6-P	Mannose-6-phosphate
MDH	Malate Dehydrogenase
FUM	Fumarate hydratase
Fum	Fumarate
Mal	Malate
α-KG	α-Ketoglutarate
GS	Glutamine Synthetase
XOD	Xanthine Oxidase
Gln	Glutamine
pGlu	Pyroglutamate
Hx	Hypoxanthine
SDH	Succinate Dehydrogenase
CCO	Cytochrome C Oxidase
GADPH	Glyceraldehyde-3-Phosphate Dehydrogenase
Ala	Alanine
Gua	Guanine
Gnt	Gluconate
SAH	S-Adenosylhomocysteine
NADH	Nicotinamide Adenine Dinucleotide
FADH_2_	Flavin Adenine Dinucleotide
OAA	Oxaloacetate
acetyl-CoA	Acetyl-Coenzyme A
Ade	Adenine
Phe	Phenylalanine
Pyr	Pyruvate
ROS	Reactive Oxygen Species
ETC	Electron Transport Chains

## Appendix A

**Table A1:** Primers of genes used for quantitative real-time PCR.

Gene Name	Gene ID	Primer (5′ to 3′)
*GAPDH*	AGABI2DRAFT_138631	F: TCATCGCATACACCGACGAG
		R: CAGCTTGACAAAGTTCGGGC
*GS*	AGABI2DRAFT_135514	F: TTGCTGTTTACGGGGAGGAC
		R: ACCGGAGCTGAAATTGCTGA
*PFK*	AGABI2DRAFT_191352	F: GTGCTAGTGCTAGTCGCGAT
		R: CAAAGCACCCATTGTTGCGA
*HK*	AGABI2DRAFT_194802	F: CACACAAGGACCCACGATGA
		R: TCCGCATTCTTTGAGGGGTC
*PK*	AGABI2DRAFT_189950	F: TGCTGTCCTTGATGGCTCTG
		R: TTTCCGCAAGCAAGCAAGTC
*SDH2*	AGABI2DRAFT_195434	F: GGGAAGGTAGCAAGGACACC
		R: GGGTTGTCGTTCTGTAGCCA
*SDH3*	AGABI2DRAFT_217734	F: AACAAACAACGCCTCCAACG
		R: TGGTTGGTGCCACGAGATAC
*SDH4*	AGABI2DRAFT_195170	F: TGCTACATGGACGTTGCGTA
		R: GCTCCGTGAGACCAATGTCA
*COX*	AGABI2DRAFT_195535	F: TGTATCTCGCCGCTGCTGTC
		R: GGAAGAGGATGAGGAATAGGTTGTTG
*MDH*	AGABI2DRAFT_187815	F: CCCAACCTCTCTACCACTGAGTATG
		R: GCCAGCGTAAGCCATAGACAAG
*CS*	AGABI2DRAFT_116105	F: CTCTCCTATGGTGCCGCTCTC
		R: AGGTTCCTCCCCGATTTTAGCC

**Table A2:** Detailed comparison of PCA and OPLS-DA models and parameters.

No. Model	Type	A	N	R2X (cum)	R2Y (cum)	Q2 (cum)
All	PCA-X	2	9	0.732		0.463
D0 VS. D18-0	OPLS-DA	1 + 1 + 0	6	0.923	0.992	0.979
D18-0 VS. D18-6	OPLS-DA	1 + 1 + 0	6	0.916	0.995	0.975

**Table A3:** D0 vs. D18-0 differential metabolites analysis table.

Metabolite	KEGG Compound ID	*m*/*z*	rt	VIP
Pyroglutamic acid	C01879	130.0496	27.1	1.174096691
S-Adenosylhomocysteine	C00021	385.1283	39.2	1.388678058
Glucose 6-phosphate	C00092	259.021	21.2	1.381533036
Fructose 6-phosphate	C00085	259.021	21.2	1.381533036
Mannose 6-phosphate	C00275	259.021	21.2	1.381533036
Malic acid	C00149	133.0134	23.7	1.147574338
Guanine	C00242	152.0564	39.4	1.382992088
Glutamine	C00064	147.0761	26.3	1.334463932
Alanine	C00041	88.0399	23.4	1.366243632
Gluconic acid	C00257	195.0497	22.1	1.316753989
Fumaric acid	C00122	115.0029	24.7	1.2064288
Pyruvaldehyde	C00546	71.0134	24.8	1.152295424
LPC (18:2/0:0)	C04100	520.3381	277.4	1.380584982
Pentadecanoic acid	C16537	241.2157	311.9	1.38873184
2-Aminobutyric acid	C02356	102.0554	23.5	1.364413792
Dimethylglycine	C01026	102.0554	23.5	1.364413792
cis-11.14-Eicosadienoic acid	C16525	307.2622	321.8	1.382379616
2-Aminoisobutyric acid	C03665	102.0554	23.5	1.364413792
Linoleic acid	C01595	279.231	309.4	1.381462975
Heptadecanoic acid	-	269.2468	325.7	1.383213561
2-Hydroxyadenine	-	152.0564	39.4	1.382992088
13(S)-HpOTrE	C04785	309.2052	258.5	1.390653128
N2, N2-Dimethylguanosine	-	312.1294	62.7	1.386101141
Phe-Pro	-	263.1386	151.8	1.357294019

**Table A4:** D18-0 vs. D18-6 differential metabolites analysis table.

Index	KEGG Compound ID	*m*/*z*	rt	VIP
Pyroglutamic acid	C01879	130.0496	27.1	1.475711527
S-Adenosylhomocysteine	C00021	385.1283	39.2	1.493191173
Glucose 6-phosphate	C00092	259.021	21.2	1.484950189
Fructose 6-phosphate	C00085	259.021	21.2	1.484950189
Mannose 6-phosphate	C00275	259.021	21.2	1.484950189
Malic acid	C00149	133.0134	23.7	1.486176097
Guanine	C00242	152.0564	39.4	1.334163806
Glutamine	C00064	147.0761	26.3	1.492244593
Alanine	C00041	88.0399	23.4	1.476759976
Gluconic acid	C00257	195.0497	22.1	1.448596129
Fumaric acid	C00122	115.0029	24.7	1.483228133
PC (8:0/8:0)	-	492.3074	261.6	1.416333102
p-Toluquinone	-	121.0287	189.4	1.455615593
Ectoine	C06231	143.0811	24.8	1.485746794
Benzoic acid	C00539	121.0287	189.4	1.455615593
Phenylalanylphenylalanine	-	313.1541	166.8	1.491385128
Valine	C00183	116.0709	24.9	1.482089512
4-(Methylamino)butanoic acid	C15987	116.0709	24.9	1.482089512
5-Aminopentanoic acid	C00431	116.0709	24.9	1.482089512
Maleic acid	C01384	115.0029	24.7	1.483228133
Phe-Leu	-	277.1542	163.4	1.492146494
2-Thiohydantoin	-	115.0029	24.7	1.483228133
